# Overexpression of KLHL23 protein from read‐through transcription of *PHOSPHO2‐KLHL23* in gastric cancer increases cell proliferation

**DOI:** 10.1002/2211-5463.12136

**Published:** 2016-10-24

**Authors:** Eun‐Seok Choi, Hanna Lee, Chang‐Hun Lee, Sung‐Ho Goh

**Affiliations:** ^1^ Precision Medicine BranchResearch InstituteNational Cancer CenterGoyangGyeonggi‐doKorea; ^2^ Department of Environmental Medical BiologyInstitute of Tropical MedicineYonsei University College of MedicineSeoulKorea; ^3^ Cancer Cell and Molecular Biology BranchResearch InstituteNational Cancer CenterGoyangGyeonggi‐doKorea

**Keywords:** gastric cancer, *PHOPHO2‐KLHL23*, read‐through transcription

## Abstract

Gene fusion, as a prototypical pathognomonic mutation, contributes to genome complexity, and the *cis*‐transcription‐induced gene fusions generated by read‐through transcription of adjacent genes have been found to be important for tumor development. We screened read‐through transcription events from stomach adenocarcinoma RNA‐seq data and selected three candidates *PHOSPHO2‐KLHL23, RPL17‐C18orf32*, and *PRR5‐ARHGAP8*, to assess their biological role in gastric cancer. The expression of all three read‐through fusion transcripts was confirmed in gastric cancer cell lines and paired normal/tumor gastric cancer tissues by real‐time quantitative reverse transcription polymerase chain reaction and their expression was found to be significantly higher in the tumor (*P* < 0.05; *n* = 75). The correlation between the expression level and clinicopathological information was statistically analyzed. The level of the *PHOSPHO2‐KLHL23* read‐through fusion transcript correlated with the Lauren classification and was significantly associated with the presence of perineural invasion. Overexpression of KLHL23 from *PHOSPHO2*‐*KLHL23* read‐through transcript led to a significant increase in cell proliferation and resistance to anticancer drug treatment. Silencing of KLHL23 expression decreased cyclin D1 levels. The expression of KLHL23 from prevalent read‐through transcripts of *PHOSPHO2‐KLHL23* in gastric cancer may undermine the efficacy of anticancer drug treatment.

**Abbreviations**
HDF: human dermal fibroblast
SRA: sequence read archives

Genetic alterations are tightly associated with the development of neoplasia. The types and patterns of such mutations are highly variable and heterogeneous within a single entity as well as between different tumor categories. Gene fusions are a prototypical example of pathognomonic mutations in tumorigenesis, and the detection and characterization of gene fusion events is of considerable importance for clinical purposes and for understanding tumorigenesis 1. Recent advances in sequencing technology have enabled a comprehensive detection of rearrangements in cancer genome and transcriptome. Gene fusion events originate predominantly from chromosomal rearrangements, which are important cancer‐associated somatic alterations 2. One of the representative examples is *BCR‐ABL1*, first reported in chronic myeloid leukemia. Identification and characterization of this fusion led to discoveries of novel therapeutics for tyrosine kinase inhibition, such as dasatinib and imatinib (Gleevec) 3.

In addition to the fusion events generated by chromosomal rearrangements, gene fusion by read‐through transcription, also known as transcription‐induced gene fusion, occurs when two of adjacent genes on the *cis*‐strand are spliced together 4. The existence of such chimeric structured transcripts has been sporadically reported before the systematic survey of human transcriptome by the ENCODE project 5, 6. In these studies, many candidate expressed sequence tags were found to cross the border of individual genes and span both genes in the pair. The frequency of the read‐through transcription events was estimated to be only 4–6% of genes in the human genome 7.

The prevailing hypothesis of the read‐through transcription mechanism is transcriptional ‘leakage’ that occurs when transcription of polymerizing RNA is not terminated at the upstream gene and proceeds to the adjacent downstream gene on a single‐strand transcript 5. Epigenetic inactivation by promoter methylation or deletion of the poly(A) transcription termination signal, which prevents proceeding of RNA polymerase II to the next gene, also results in read‐through transcription in cancer 8, 9. When a read‐through transcript is spliced into a gene fusion, it follows several intergenic splicing patterns, and most of them (44%) occur between two exons, exon (*n* − 1) (one before the last) from the proximal gene and the exon 2 of the distal gene 10. Therefore, the fusion transcripts can pass the termination of translation, when they are engaged in the ribosome machinery.

These kinds of variations in the transcriptome are the source of generating diversity of proteins translated from a finite number of genes in the genome, and a mechanism contributing to the evolution of protein complexities. In certain cases, read‐through transcription is translated into functional protein products that could contribute to the progression of diseases, especially to tumor development. For example, *CUL3‐KLHL7* attenuation is related to a retinitis pigmentosa causative mutation 11, *SLC45A3‐ELK4* expression level correlates with prostate cancer progression 12, *YPEL5‐PPP1CB* is involved in the development of chronic lymphocytic leukemia 13. Aberrant read‐through transcription has been also reported to contribute to the formation of solid tumors in colorectal cancer 14, 15, renal cell carcinoma 16, bladder cancer 17, and breast cancer 18. However, there have been no reports about the read‐through transcription profiles in gastric cancer.

The biological functions of the read‐through fusion gene components analyzed in this study have not been characterized with respect to their possible tumorigenic role. For example, the expression of *KLHL23* (Kelch‐Like Family Member 23) during tumorigenesis in cancer has not been previously evaluated, although its association with cone‐rod dystrophy has been reported 19. The function of *PHOSPHO2* (Phosphatase, Orphan 2) has been attributed to the vitamin B6 pathway, but not to any tumorigenic phenomenon 20.

In this study, we screened the fusion gene databases and selected read‐through transcription‐induced chimeras detected in gastric tissues. Among those, the read‐through transcripts that included proteins whose biological properties could be relevant to oncogenic activity were validated in tissue samples from patients with gastric cancer and analyzed further.

## Materials and methods

### Database search of read‐through transcription candidates

For the screening of read‐through fusion transcripts from gastric cancer tissues, totally 42 sequence read archives (SRA) files (raw data files) for RNA‐seq were selected for 30 gastric tissues and 12 stomach cancer cell lines and downloaded from NCBI SRA archive and European Nucleotide Archives of EMBL‐EBI. These data were analyzed to generate fusion candidate using fusion detection software, including tophat fusion 21 and fusionq, 22 but excluded false‐positive detection with low complexity. The Human genome build hg.19 was used as a reference genome for this analysis. We also searched fusion gene candidates from COSMIC 23 and ConjoinG databases (http://metasystems.riken.jp/conjoing/index).

### Cell lines and fresh human tissue samples

In this study, we used AGS, MKN‐28, KATO‐III, and human dermal fibroblast (HDF) cell lines obtained from American Type Culture Collection (Manassas, VA, USA), and SNU‐638 and SNU‐216 cell lines from Korea Cell Line Bank (Seoul, Korea). All the cells were cultured with designated media (Cellgro; Corning, Manassas, VA, USA) supplemented with 10% FBS serum (Cellgro; Corning) and 1× Penicillin Streptomycin (Invitrogen, Carlsbad, CA, USA) at 37 °C containing 5% CO_2_.

Fresh human stomach tissues were obtained from patients who agree the informed consent approved by institutional review board of National Cancer Center of Korea (NCCNCS13732; Table 1). Dissected tissues were stored in RNA later solution (Qiagen, Hilden, Germany) for total RNA extraction or liquid nitrogen for protein extraction.

**Table 1:** Clinicopathological information of gastric cancer patients for mRNA expression‐level assessment between tumor and normal tissues (*n* = 75)

	*n*
Number of patients	
Total	75
Male	53
Female	22
Age at diagnosis (years)	
Range	21–86
Mean ± SD	60.7 ± 13.3
Disease stage	
Tumor stage	
T1a	0
T1b	1
T2	16
T3	29
T4a	22
T4b	7
Node stage	
N0	26
N1	11
N2	13
N3a	17
N3b	8
Metastasis stage	
M0	63
M1	12
Lauren type	
Intestinal	33
Diffuse	25
Mixed	7
Indeterminate	8
Not annotated	2
Borrmann type	
Type I (protruded type)	1
Type II (ulcerative type)	21
Type III (ulceroinfiltrative type)	46
Type IV (diffuse type)	6
Not determined	1
Invasion	
Perineural	
Present	37
Not identified	38
Venous	
Present	12
Not identified	63

### RT‐PCR and quantitative RT‐PCR

Total RNA was extracted using Trizol reagent (Invitrogen) according to the manufacturer's protocol and purified with RNeasy column with RNase‐free DNaseI treatment (Qiagen). We used one microgram of total RNA to synthesize first‐strand cDNA primed with poly‐d(T)_18–21_ primers using Transcriptor cDNA synthesis kit (Roche Applied Science, Basel, Switzerland). We used 10 ng of cDNA per 20 μL reaction for the end‐point RT‐PCR or real‐time PCR.

Primers and probes for RT‐PCR or qRT‐PCR were designed using primer3 software 24 to span two consecutive exons (Table S1). qRT‐PCR reactions were performed using a LightCycler 480 (Roche Applied Science); results were quantitated by standard curve‐based absolute quantitation.

### Assessments of read‐through fusion transcript effect on tumorigenesis

*PHOSPHO2‐KLHL23* expression was silenced in AGS gastric cancer cells by transfecting with *KLHL23*‐specific siRNA (SI04213391 and SI03205321; Qiagen). Allstar negative control (NC) siRNA (SI03650318; Qiagen) was used as a negative control. Cells, plated in six‐well culture plates (8 × 10^4^ cells/well), were transfected with 5 nm siKLHL23 or NC siRNA using HiPerFect transfection reagent (301707; Qiagen) according to the manufacturer's protocol.

Cell proliferation assay were performed using cells seeded at a density of 5 × 10^3^ cells per well in 96‐well plates and treated with etoposide (Sigma‐Aldrich, St. Louis, MO, USA) at the indicated concentrations. After incubation for 24 h, the washed cells were incubated in 100 μL of MTT [3‐(4,5‐dimethylthiazol‐2‐yl)‐2,5‐diphenyl tetrazolium bromide, 0.5 mg·mL^−1^] at 37 °C for 4 h in a 5% CO_2_ environment. The absorbance at 450 nm was measured using a VersaMax^™^ Microplate Reader (Molecular Devices, Sunnyvale, CA, USA).

### Immunoblot analyses

The lysates were denatured for 10 min in SDS sample buffer and separated by SDS/PAGE followed by electroblotting onto polyvinylidene difluoride membrane. After blocking of 3% skim milk, the membranes were incubated with ACTB (A1978; Sigma‐Aldrich), KLHL23 (sc‐137551; Santa Cruz Biotechnology, Dallas, TX, USA), p21 (#2947, Cell Signaling Technologies, Danvers, MA, USA) and Cyclin D1 (#2978, Cell Signaling Technologies) followed by HRP‐conjugated secondary antibody.

### Statistical analysis

The statistical significance of differences between groups was determined using Student's *t*‐test. *P*‐values less than 0.05 were considered statistically significant.

## Results

### Screening of the read‐through transcription candidates

We searched for the read‐through transcription candidates using stomach adenocarcinoma RNA‐seq data from public databases. All candidates were thoroughly collected from various bioinformatic databases (UCSC archive, EMBL‐EBL ENA archive, NCBI SRA archive, Cosmic database, ConjoinG database) using fusion detection tools. We excluded false‐positive results and obtained 83 candidates from bioinformatic screening (Table S2). Among the candidates, we focused on three read‐through transcription candidates that included gastric cancer‐related genes (*PHOSPHO2‐KLHL23*,* RPL17‐C18orf32*,* PRR5‐ARHGAP8*). Their presence in the stomach tissues has been annotated in public databases.

### Verification of the read‐through fusion transcripts in gastric cancer cell lines

**Figure 1:**
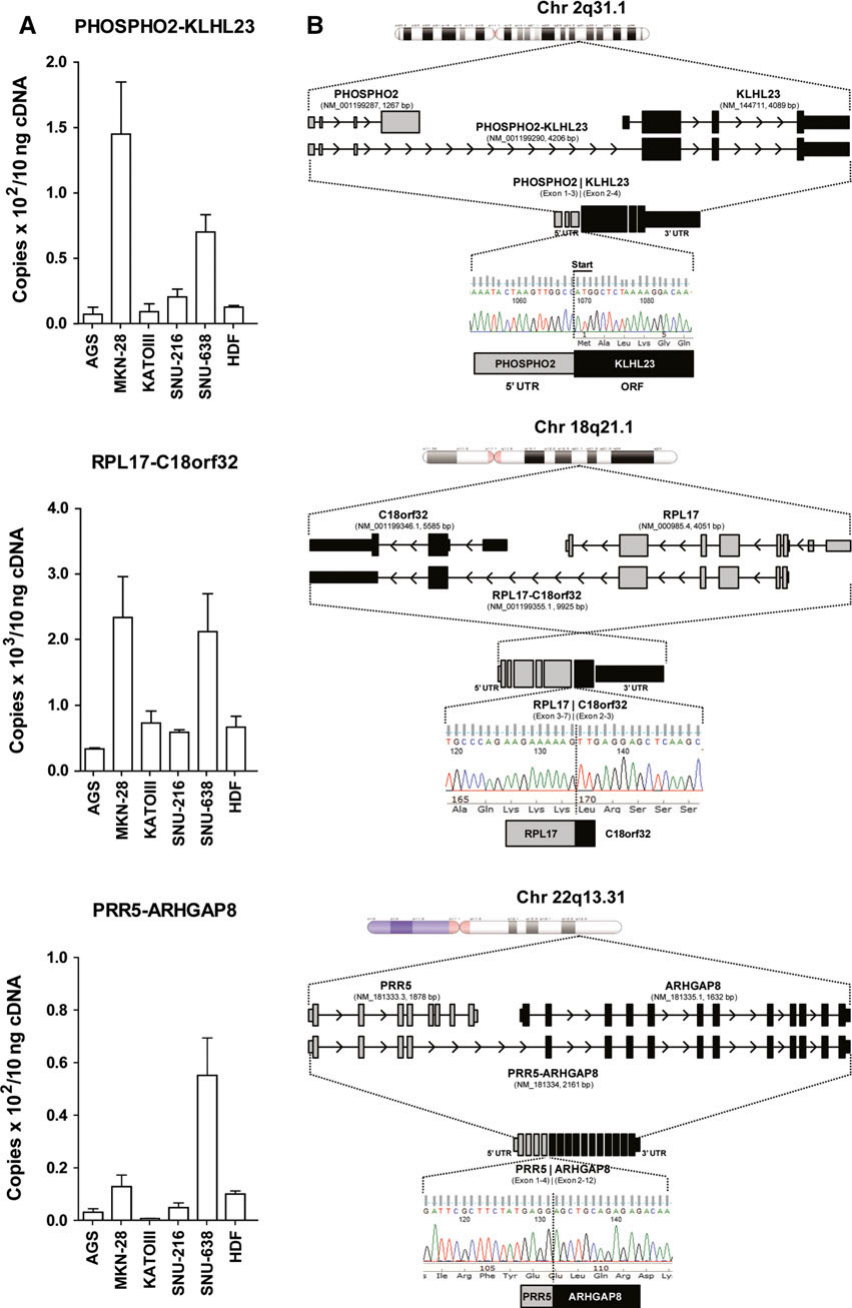
Verification of read‐through fusion transcripts in gastric cancer cell lines. (A) The expression level of three read‐through fusion transcripts was measured with absolute quantitation by qRT‐PCR in gastric cancer cell lines. (B) Genomic alignment of sequenced read‐through fusion transcripts from MKN‐28 gastric cancer cell line. Chromosome location, gene order, read‐through fusion transcript, and fusion protein structure were illustrated. The fusion junction of read‐through transcript was depicted by DNA sequence chromatogram. Gray box indicates proximal gene and black box indicates distal gene.

To confirm the relevance of these read‐through fusion transcripts to gastric cancer, we carried out RT‐PCR analysis of five gastric cancer cell lines (AGS, MKN‐28, KATO‐III, SNU‐216, and SNU‐638) and the nongastric HDF cancer cell line. *PHOSPHO2‐KLHL23*,* RPL17‐C18orf32*, and *PRR5‐ARHGAP8* transcripts were robustly detected in all cell lines (Fig. 1A). *PHOSPHO2‐KLHL23* and *RPL17‐C18orf32* were highly expressed in the MKN‐28 and SNU‐638 cell lines. *PRR5‐ARHGAP8* showed high expression in the SNU‐638, but not in MKN‐28 cells. We confirmed the junction between the proximal and distal genes in the read‐through fusion transcripts by sequencing and found that all three read‐through fusion transcripts of our interest skipped exon 1 of the distal gene (Fig. 1B). Interestingly, most read‐through fusions could be translated into fused proteins, whereas *PHOSPHO2‐KLHL23* translated only *KLHL23*, because one ORF was skipped in *PHOSPHO2*.

### Differential expression of the read‐through fusion transcripts in stomach tumor tissues and correlation with clinicopathological parameters

**Figure 2:**
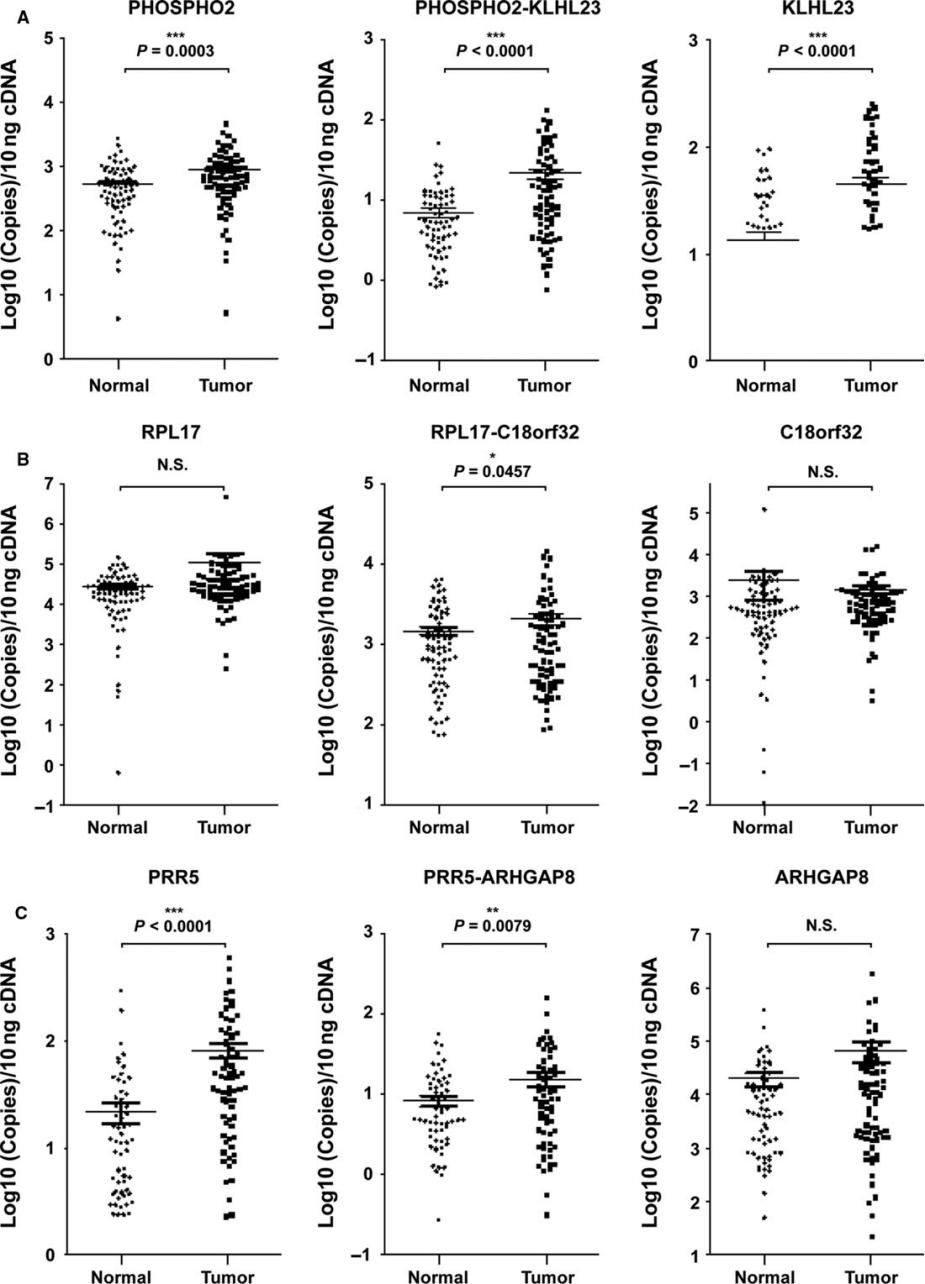
Quantitation of mRNA expression levels of read‐through fusion transcripts in 75 advanced gastric cancer patient tissues. (A) Quantitation of mRNA level of *PHOSPHO2*,* PHOSPHO2‐KLHL23*, and *KLHL23*. (B) Quantitation of mRNA level of *RPL17*,* RPL17‐C18orf32*, and *C18orf32*. (C) Quantitation of mRNA level of distal gene of read‐through fusion transcripts: *PRR5*,* PRR5‐ARHGAP8*, and *ARHGAP8*. The expression of read‐through fusion transcripts was detected at junction region between two genes. Primer pairs anchor on each side of proximal and distal gene, and a junction region‐specific Taqman probe was applied to enhance sequence specificity (*P < 0.05, **P < 0.01, ***P < 0.001; N.S., not significant).

To test whether the expression level of the read‐through fusion transcripts differed in patients, we measured absolute mRNA expression by quantitative RT‐PCR in normal and tumor tissues of 75 advanced gastric cancer patients (Table 1). The expression levels of the *PHOSPHO2‐KLHL23* read‐through fusion transcript as well as its proximal and distal genes were significantly elevated in patient tumor tissues (Fig. 2A). The *RPL17‐C18orf32* read‐through transcript was significantly upregulated in tumors, whereas its proximal and distal genes were not differentially expressed (Fig. 2B). The expression levels of *PRR5‐ARHGAP8* and its proximal gene were also higher in tumor samples than in normal tissues, whereas its distal gene expression was not elevated (Fig. 2C). We analyzed the correlation of the levels of the read‐through fusion transcripts with several clinicopathological parameters, and found that *PHOSPH2‐KLHL23* expression significantly correlated with the Lauren classification (*P* = 0.040) and perineural invasion (*P* = 0.037), whereas no correlations were observed for the *RPL17‐C18orf32* and *PRR5‐ARHGAP8* fusion transcripts (Table 2).

**Table 2:** Statistical analyses of read‐through fusion transcript expression with clinicopathological parameters

Class	*PHOSPHO2‐KLHL23*		*RPL17‐C18orf32*		*PRR5‐ARHGAP8*	
	Mean	*P*‐value	Mean	*P*‐value	Mean	*P*‐value
Lauren classification						
Intestinal	6.03	0.040	2.21	0.208	4.27	0.054
Diffuse	2.56		1.66		1.34	
Histology						
Well diff.	4.85	0.672	1.98	0.537	6.74	0.145
Moderate diff.	3.54		1.48		2.37	
Poorly diff.	5.24		1.55		0.95	
Bormann type						
Bormann 1	3.46	0.055	1.75	0.697	3.72	0.065
Bormann 2	10.39		2.52		7.45	
Bormann 3	3.95		1.67		1.60	
Bormann 4	0.84		1.04		0.64	
Not identified	1.52		2.27		1.60	
pT stage						
T1b	1.52	0.860	2.27	0.726	1.60	0.766
T2	6.43		1.81		4.11	
T3	5.00		2.25		3.05	
T4a	3.62		1.53		1.72	
T4b	5.30		0.97		0.98	
pN stage						
N0	6.90	0.515	2.43	0.376	4.94	0.532
N1	3.27		1.40		2.13	
N2	3.63		1.53		1.64	
N3a	5.43		2.04		1.76	
N3b	2.19		0.58		1.98	
Invasion—venous						
Present	6.27	0.224	1.53	0.201	1.57	0.083
Not identified	4.70		1.89		3.00	
Invasion—perineural						
Present	3.21	0.037	1.40	0.065	1.55	0.067
Not identified	6.57		2.26		4.01	

### Expression of the read‐through transcript *PHOSPHO2‐KLHL23* correlates with enhanced cell proliferation

**Figure 3:**
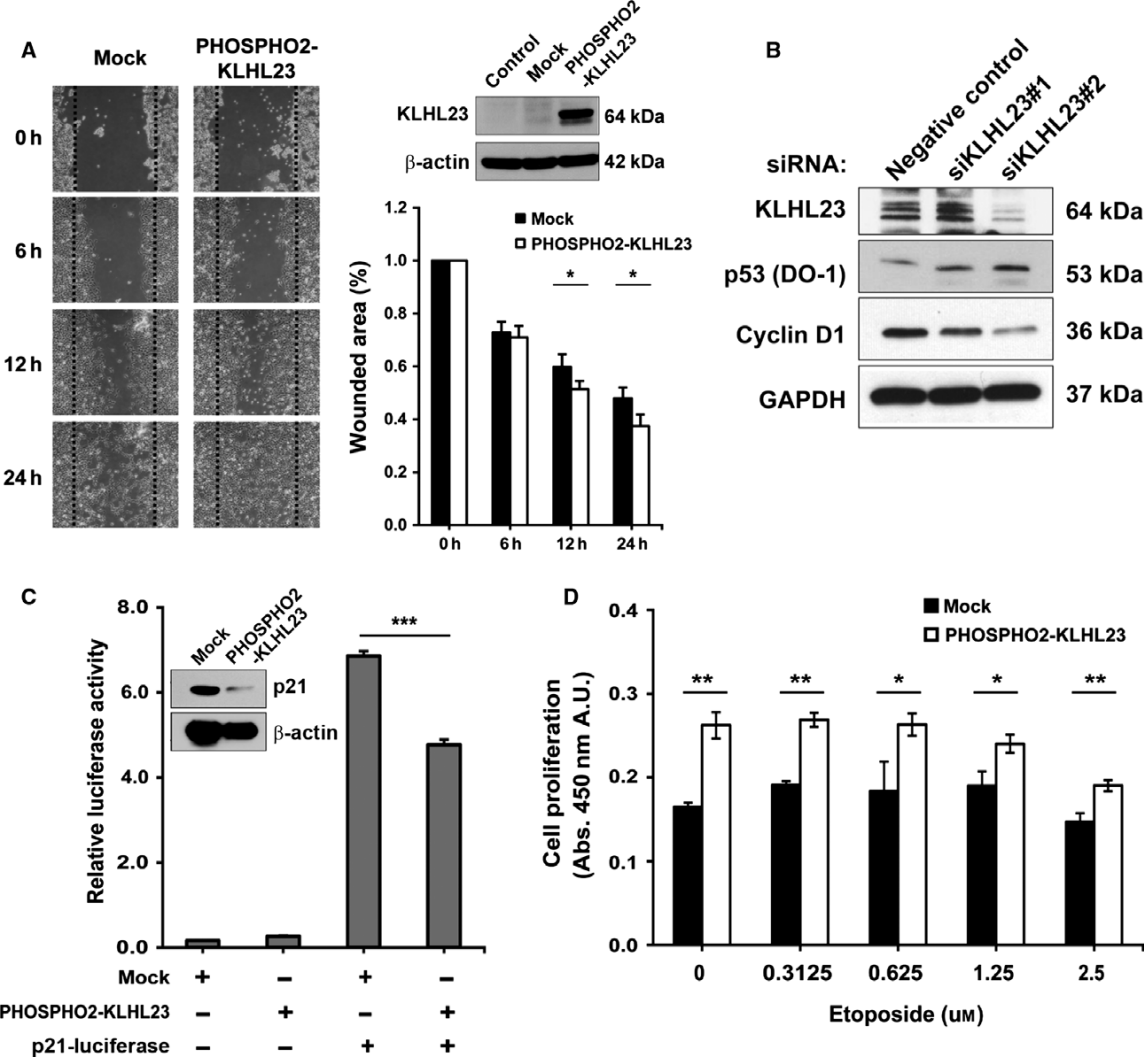
Biological functions of PHOSPHO2‐KLHL23 associated with cell proliferation. (A) *PHOSPHO2‐KLHL23* stable transfectant in HEK‐293 cells showed significantly higher wound recover at 12 and 24 h. (B) Knockdown of KLHL23 with the treatment of siRNA induction of cell death protein p53 in AGS cell. On the contrary, it decreased the expression level of cyclin D1. (C) Decrement of p21 protein expression by *PHOSPHO2‐KLHL23* overexpression in HEK‐293 cells (inset). Dual‐luciferase assay also showed that *PHOSPHO2‐KLHL23* downregulated p21 promoter activity. (D) The *PHOSPHO2‐KLHL23* stable transfectant is more resistant to etoposide treatment (< 2.5 μm).

Among the read‐through fusion transcript candidates, only *PHOSPHO2*‐*KLHL23* was characterized by a significant correlation of its expression with clinicopathological parameters (Table 2). We postulate that the increased *PHOSPHO2*‐*KLHL23* expression may be related to tumorigenic processes. To examine whether the tumorigenic ability of tumor tissues is augmented by the increased expression of *PHOSPHO2*‐*KLHL23*, we carried out a proliferation assay using HEK‐293 cells that stably expressed the *PHOSPHO2‐KLHL23* construct. However, although the mRNA transcript comprised the whole read‐through fusion sequence, the open reading frame of this construct enabled translation of the *KLHL23* gene only, and not of the whole *PHOSPHO2*‐*KLHL23*‐fused transcript. Nonetheless, the stable expression of *PHOSPHO2‐KLHL23* in HEK‐293 cells closed the wounded area relatively faster at 12 and 24 h in wound healing assay (Fig. 3A).

Next, to explore the link between *KLHL23* expression from the *PHOSPHO2‐KLHL23* read‐through fusion and the extent of cell growth, we monitored levels of the KLHL23, p53, and cyclin D proteins after silencing *KLHL23* expression in AGS cell line. This cell line expresses the read‐through fusion transcript of *PHOSPHO2*‐*KLHL23*, as well as the corresponding proximal and distal genes (Fig. 1 and Fig. S1). We tested two *KLHL23* siRNA and only siKLHL23#2 had a proper *KLHL23* silencing effect. Knockdown with *KLHL23* siRNA (siKLHL23#2) led to the downregulation of cyclin D1 and upregulation of p53 (Fig. 3B). We further characterized the effect of *PHOSPHO2‐KLHL23* in an opposite way by overexpressing this construct. This manipulation decreased the level of the p21 protein (Fig. 3C inset). Dual‐luciferase assay of the p21 promoter performed in HEK‐293 cells transfected with the *PHOSPHO2‐KLHL23* expression construct also showed a significant decrement in p21 promoter activity (Fig. 3C). These results suggest that increased *PHOSPHO2‐KLHL23* expression is associated with cell proliferation and/or cell cycle events, and that upregulation of the *PHOSPHO2‐KLHL23* read‐through fusion transcript has a tumorigenic effect. The effect of the PHOSPHO2‐KLHL23‐fused protein on drug sensitivity was measured at several concentrations of etoposide, a DNA‐damaging agent. The proliferation of cell lines stably expressing *PHOSPHO2‐KLHL23* was significantly higher at low concentrations of the drug (< 2.5 μm) compared to the proliferation of untreated control cells at 24 h (Fig. 3D). However, at higher concentrations of etoposide (over 5 μm), the proliferation of cell lines stably expressing *PHOSPHO2‐KLHL23* did not differ from that of control cells (data not shown).

## Discussion

With the increase in the number of available RNA‐seq datasets from various cancer tissues, the amount of evidence about read‐through transcripts is rapidly growing. It is possible that upregulation of the read‐through fusion transcripts may correlate with the development of cancer, although downregulation is implicated in lung adenocarcinoma 25. To confirm this, we screened stomach adenocarcinoma transcriptome data and selected three candidate read‐through fusion transcripts. Analysis of stomach cancer patient tissues as well as of several cancer cell lines showed robust expression of all three read‐through fusion transcripts (Figs 1 and 2). In particular, changes in *PHOSPHO2‐KLHL23* mRNA expression were the most significant. The analysis of the relationship between expression differences and clinicopathological parameters revealed that *PHOSPHO2‐KLHL23* mRNA expression correlated with perineural invasion (Table 2).

To assess tumorigenic implications of the read‐through fusion activity, we first considered biological functions of each proximal and distal gene product. As revealed by the analysis of gene ontology database data, their functions were closely related to cell growth and survival. PHOSPHO2 is important for metabolism and the vitamin B6 metabolism pathway 26, and KLHL23 is implicated in cone‐rod dystrophy and the vitamin B6 metabolism pathway 27. Ribosomal protein L17 (RPL17) also known as RPL23 is a component of the large 60S ribosome subunit and it promotes multidrug resistance in gastric cancer cell by suppressing drug‐induced apoptosis 28, 29. The chromosome 18 open reading frame 32 (C18ORF32) protein possibly activates the NF‐kappa‐B signaling pathway 30. Proline rich 5 [PRR5 (Renal)] is a component of the mammalian target of rapamycin complex 2 (mTORC2) that regulates platelet‐derived growth factor (PDGF) receptor beta expression and PDGF signaling to Akt and S6K1 31. Rho GTPase‐activating protein 8 (ARHGAP8) encodes a member of the RHOGAP family and is found to be overexpressed in primary colorectal tumors 32. Among these candidates, we assessed biological functions of the most significantly altered read‐through fusion transcript, *PHOSPHO2‐KLHL23*. When this transcript is translated, only the distal gene protein product KLHL23 is generated. Notably, as already mentioned, this protein has not been characterized for its role in tumorigenesis yet, although its dysregulation has been implicated in cone‐rod dystrophy. In this study, we assessed the effect of KLHL23 on cell proliferation by introducing the PHOSPHO2‐KLHL23 expression construct into HEK‐293 cells and measuring wound healing. The presence of *PHOSPHO2‐KLHL23* significantly affected wound healing (Fig. 3A). It also affected cell survival after treatment with a DNA‐damaging agent. Bioinformatic analysis suggested that the KLHL23 protein translated from the *PHOSPHO2‐KLHL23* transcript may modulate the activity of the cell death pathway proteins, and this was confirmed by the results of *KLHL23* knockdown (Fig. 3B, C). These results represent a potential therapeutic target to perineural invasion in gastric cancer and also might inhibit the proliferation by KLHL23 mRNA‐specific impairment in the tumor.

The read‐through transcriptions are frequently observed in tumor transcriptome‐profiling studies 33, 34. Although the mechanism for it has not been clearly elucidated, promoter methylation or missing of poly(A) tail, which prevents the proceeding of RNA polymerase II to the next gene, may give rise to read‐through transcription in cancer 8, 9. However, it is known that promoter hypermethylation causes downregulation or complete inhibition of transcription 32, 35, 36. Therefore, assuming that the difference in the promoter methylation status of consecutive genes leads to increased levels of the read‐through fusion transcripts, we retrieved information about the methylation status of promoters of the read‐through fusion transcripts as well as their proximal and distal genes from MethHC (database of DNA methylation and gene expression in Human Cancer) 37 that contains data on gene methylation and expression profiles in 18 human cancers. In addition, we analyzed 6548 microarray methylation datasets in the Cancer Genome Atlas. Methylation of the PHOSPHO2‐KLHL23 promoters was significantly reduced in tumor tissues. However, methylation profiles of the two other read‐through fusion transcripts showed different patterns (Fig. S2). These differences suggested that at least for *PHOPSPHO2‐KLHL23* read‐through transcription, the increased methylation of KLHL23 promoter in tumor tissue may block the KLHL23 transcription, which would promote read‐through transcription by PHOPHO2. Thus, more studies, such as the effects of deletion of poly (A) sequence or epigenetic changes in histone proteins on read‐through fusion, are required to elucidate the detailed mechanisms resulting in read‐through transcription 9, 38.

The list of transcript variants derived from massive transcriptome profiles is increasing and it would lead to the discovery of a novel biomarker to support traditional pathological diagnosis of tumor. Therefore, the statistical significant correlation between read‐through transcript levels in tumor and clinicopathological parameters is critical. Although this study provided partial functional characterization of *PHOSPHO2‐KLHL23*, its significant correlation with perineural invasion may be able to be used as a molecular signature to diagnose gastric cancer.

## Conclusion

In this study, we screened gastric cancer tissue samples and confirmed frequent expression of read‐through transcripts in them. Of note, *PHOSPHO2‐KLHL23* was the most significantly upregulated transcript in stomach tumor tissues and our investigation revealed that the KLHL23 protein is related to the tumorigenic effect. Our observations suggest that the existence of prevalent read‐through fusion transcripts adds a further level of complexity to tumor development, and further research is necessary to provide more comprehensive information about the role of this phenomenon in tumorigenesis.

## Author contributions

SHG conceived and designed the experiments; ESC, HL, and CHL performed the experiments; ESC, HL, CHL, and SHG analyzed the data; SHG and ESC wrote the manuscript.

## Supporting information


**Fig. S1.** Quantitation of proximal and distal gene expression at mRNA level by qRT‐PCR in stomach cancer cell lines (AGS, MKN‐28, KATOIII, SNU‐216, and SNU‐638) and noncancer cell lines (HDF and HEK‐293).
**Fig. S2.** Methylation status of read‐through fusion and distal gene promoter assessed of *PHOSPHO2‐KLHL23*.
**Table S1.** PCR primers for qRT‐PCR.
**Table S2.** Candidate read‐through transcriptions screened from gastric cancer.
