# Supplementary material for: Overexpression of KLHL23 protein from read‐through transcription of PHOSPHO2‐KLHL23 in gastric cancer increases cell proliferation
Source: FEBS Open Bio. 2016 Oct 24;6(11):1155–64. doi: 10.1002/2211-5463.12136 (PMC5095152; doi:10.1002/2211-5463.12136)
Supplement: Supplementary file 1 — Fig. S1. Quantitation of proximal and distal gene expression at mRNA level by qRT‐PCR in stomach cancer cell lines (AGS, MKN‐28, KATOIII, SNU‐216, and SNU‐638) and noncancer cell lines (HDF and HEK‐293). Fig. S2. Methylation status of read‐through fusion and distal gene promoter assessed of PHOSPHO2‐KLHL23. Table S1. PCR primers for qRT‐PCR. Table S2. Candidate read‐through transcriptions screened from gastric cancer. [file FEB4-6-1155-s001.docx]

**
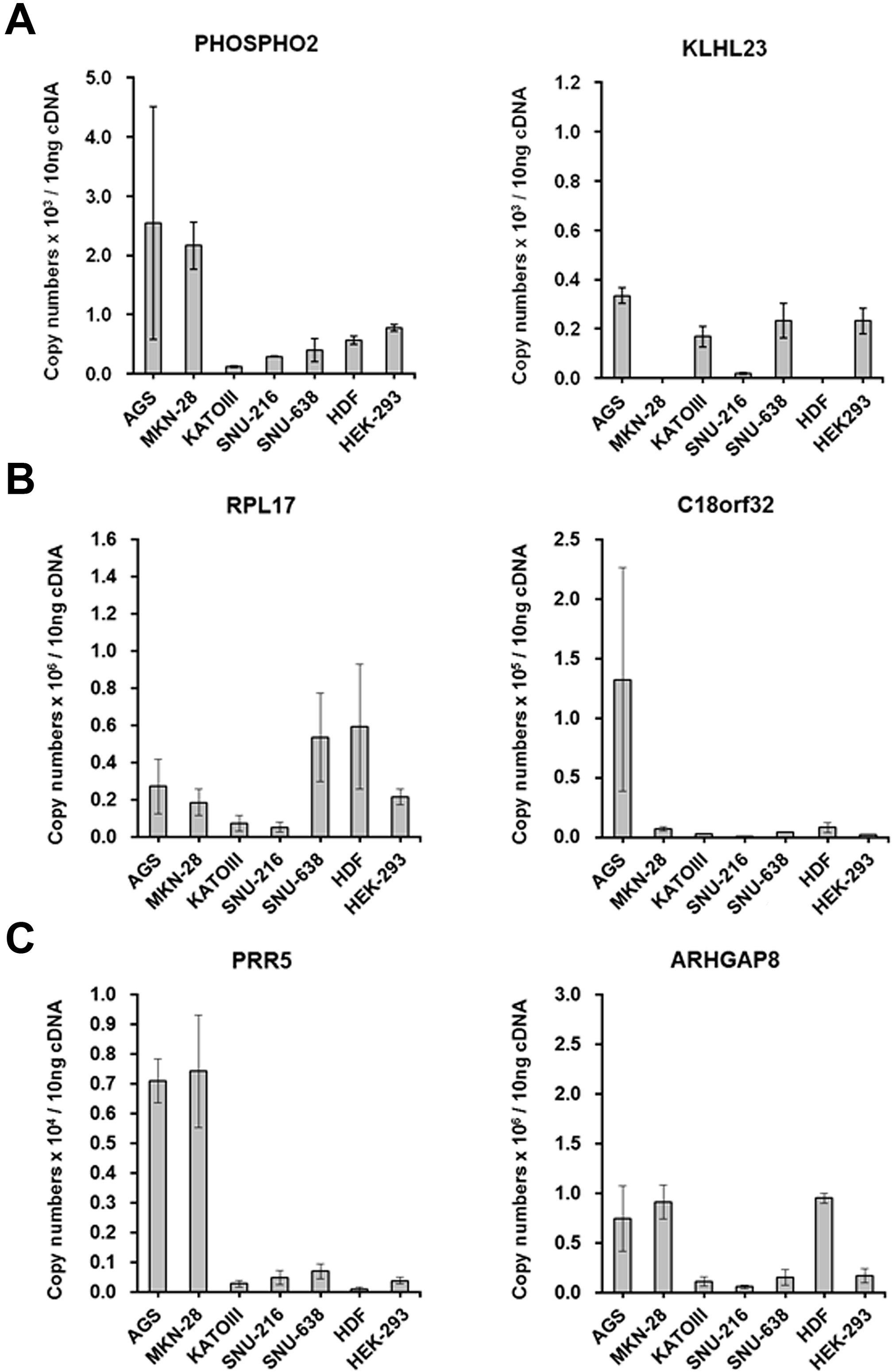
**

**Supplementary Fig. 1.** Quantification of proximal and distal gene expression at mRNA level by qRT-PCR in stomach cancer cell lines (AGS, MKN-28, KATOIII, SNU-216, and SNU-638) and non-cancer cell lines (HDF and HEK-293). (A) mRNA level of *PHOSPHO2* and *KLHL23*. (B) mRNA level of *RPL17* and *C18orf32*. (C) mRNA level of *PRR5* and *ARHGAP8*.





**Supplementary Fig. 2.** Methylation status of read-through fusion and distal gene promoter assessed of *PHOSPHO2-KLHL23*. (A), *RPL17-C18orf32* (B), and *PRR5-ARHGAP8* (C) from MethHC database. (*p < 0.05, **p < 0.01, ***p < 0.001)

**Supplementary Table 1**. PCR primers for qRT-PCR

| **Target name** | **Forward primer (5’ – 3’)**  **Reverse primer (5’ – 3’)** | **Amplicon size (bp)** | **Annealing temp. (°C)** |
| --- | --- | --- | --- |
| *ACTB* | CAT CGA GCA CGG CAT CGT CA | 211 | 58 |
|  | TAG CAC AGC CTG GAT AGC AAC |  |  |
|  |  |  |  |
| *PHOSPHO2-KLHL23* | TCC TAA TCC CAA TGC TGA AG | 125 | 58 |
|  | TGC ATC CAG AAA ATC CAC TG |  |  |
|  |  |  |  |
| *KLHL23* | AAT AGG AGC TGC TTG TGG GG | 207 | 58 |
|  | ACT GGA TGT GTT GAA TCC TTG AA |  |  |
|  |  |  |  |
| *PHOSPHO2* | ATG TGC TCC CAA CAA AAA GC | 157 | 58 |
|  | TGG AGT GAA AGG CAA TGA TG |  |  |
|  |  |  |  |
| *RPL17-C18orf32* | TCT CCC TGC CAC ATT GAG AT | 122 | 58 |
|  | AGG AAT GCA CAC CAT TTT CC |  |  |
|  |  |  |  |
| *RPL17* | TCA TGG TCG GAT TAA CCC ATA | 155 | 58 |
|  | CCC GTG CCA TAA GTT TTT GT |  |  |
|  |  |  |  |
| *C18orf32* | GAC ATT GTC AGC TGC GTT TC | 236 | 58 |
|  | CCA TTC ATG TCT GCA CCC TTA |  |  |
|  |  |  |  |
| *PRR5-ARHGAP8* | GTC CTT CTT CAC GGA GTA CCT | 144 | 58 |
|  | GAG GGC CTC TTC CTC ACT G |  |  |
|  |  |  |  |
| *PRR5* | GAC GTG TTG TCA CGT TCA GC | 230 | 58 |
|  | CCT GGG CCA CAT AGT GAG AT |  |  |
|  |  |  |  |
| *ARHGAP8* | GTG ACC TGG GCA AGA GAG AG | 225 | 58 |
|  | GCT GGT TCT GCA GGT ACT CC |  |  |

**Supplementary Table 2**. Candidate read-through transcriptions screened from gastric cancer

**A**. Read-through transcriptions from fusion detection tools

| **Chromosome** | **Position 1** | **Position 2** | **Proximal gene** | **Distal gene** |
| --- | --- | --- | --- | --- |
| chr1 | 72,639,027 | 73,322,873 | NEGR1 | HY209415 |
| chr1 | 204,381,176 | 207,080,449 | PIK3C2B | FAIM3 |
| chr11 | 61,158,722 | 62,419,842 | TMEM216 | INTS5 |
| chr16 | 23,813,094 | 28,834,251 | PRKCB | NPIPL1 |
| chr2 | 159,327,037 | 159,331,610 | CCDC148 | PKP4 |
| chr2 | 45,169,186 | 45,500,575 | SIX3 | LINC01121 |
| chr22 | 38,943,064 | 39,863,661 | DMC1 | MGAT3 |
| chr4 | 96,338,648 | 99,104,383 | UNC5C | STPG2 |
| chr4 | 164,077,263 | 166,973,329 | NAF1 | TLL1 |
| chr5 | 170,755,093 | 180,527,923 | NPM1 | BTNL9 |
| chr5 | 171,206,868 | 171,417,134 | SMIM23 | FBXW11 |
| chr5 | 145,444,132 | 146,167,067 | SH3RF2 | PPP2R2B |
| chr9 | 37,078,360 | 37,443,501 | LOC100506710 | ZBTB5 |
| chrX | 1,706,886 | 1,854,532 | AKAP17A | ASMT |
| chrX | 18,735,181 | 18,809,786 | RS1 | PPEF1 |

**B**. Read-through transcriptions from UCSC database searches

| **Chromosome** | **Position 1** | **Position 2** | **Proximal gene** | **Distal gene** |
| --- | --- | --- | --- | --- |
| chr5 | 139,889,222 | 139,929,163 | ANKHD1 | EIF4EBP3 |
| chr1 | 10,490,159 | 10,512,060 | APITD1 | CORT |
| chrX | 101,854,276 | 101,972,661 | ARMCX5 | GPRASP2 |
| chr3 | 9,834,232 | 9,896,822 | ARPC4 | TTLL3 |
| chr14 | 23,775,971 | 23,795,394 | BCL2L2 | PABPN1 |
| chr13 | 103,459,496 | 103,528,351 | BIVM | ERCC5 |
| chr15 | 90,373,831 | 90,456,222 | C15orf38 | AP3S2 |
| chr7 | 139,025,878 | 139,108,203 | C7orf55 | LUC7L2 |
| chr8 | 67,579,787 | 67,774,257 | C8orf44 | SGK3 |
| chr13 | 36,742,345 | 36,871,992 | CCDC169 | SOHLH2 |
| chr14 | 65,482,301 | 65,529,373 | CHURC1 | FNTB |
| chr16 | 66,586,466 | 66,613,038 | CKLF | CMTM1 |
| chr10 | 22,605,312 | 22,620,414 | COMMD3 | BMI1 |
| chr16 | 4,411,691 | 4,466,962 | CORO7 | PAM16 |
| chr7 | 99,282,302 | 99,332,819 | CYP3A7 | CYP3AP1 |
| chr9 | 114,393,632 | 114,432,526 | DNAJC25 | GNG10 |
| chr20 | 44,164,919 | 44,176,065 | EPPIN | WFDC6 |
| chr10 | 50,723,151 | 50,747,169 | ERCC6 | PGBD3 |
| chr4 | 77,172,853 | 77,232,283 | FAM47E | STBD1 |
| chr1 | 74,663,896 | 75,010,116 | FPGT | TNNI3K |
| chr11 | 117,690,790 | 117,747,746 | FXYD6 | FXYD2 |
| chr7 | 150,413,645 | 150,440,737 | GIMAP1 | GIMAP5 |
| chr2 | 198,364,721 | 198,418,423 | HSPE1 | MOB4 |
| chr11 | 2,167,853 | 2,182,439 | INS | IGF2 |
| chr3 | 159,481,930 | 159,612,500 | IQCJ | SCHIP1 |
| chr3 | 128,806,412 | 128,880,073 | ISY1 | RAB43 |
| chr15 | 42,120,283 | 42,140,346 | JMJD7 | PLA2G4B |
| chr12 | 10,524,952 | 10,560,388 | KLRC4 | KLRK1 |
| chr2 | 160,625,139 | 160,761,267 | LY75 | CD302 |
| chrX | 151,282,521 | 151,307,050 | MAGEA10 | MAGEA5 |
| chr19 | 19,256,376 | 19,293,492 | MEF2BNB | MEF2B |
| chr1 | 19,923,471 | 19,984,949 | MINOS1 | NBL1 |
| chr9 | 103,204,188 | 103,339,918 | MSANTD3 | TMEFF1 |
| chr11 | 77,726,761 | 77,791,265 | NDUFC2 | KCTD14 |
| chr14 | 24,682,615 | 24,701,576 | NEDD8 | MDP1 |
| chr17 | 49,230,897 | 49,249,105 | NME1 | NME2 |
| chr2 | 18,735,989 | 18,770,846 | NT5C1B | RDH14 |
| chr9 | 112,542,577 | 112,900,819 | PALM2 | AKAP2 |
| chr2 | 170,550,964 | 170,608,396 | PHOSPHO2 | KLHL23 |
| chr1 | 156,182,779 | 156,207,082 | PMF1 | BGLAP |
| chr12 | 89,913,190 | 89,920,039 | POC1B | GALNT4 |
| chr19 | 10,216,899 | 10,226,064 | PPAN | P2RY11 |
| chr22 | 45,098,078 | 45,258,664 | PRR5 | ARHGAP8 |
| chr17 | 41,102,543 | 41,132,545 | PTGES3L | AARSD1 |
| chr7 | 5,023,302 | 5,112,854 | RBAK | RBAKDN |
| chr11 | 66,384,053 | 66,413,944 | RBM14 | RBM4 |
| chr2 | 86,730,553 | 86,948,245 | RNF103 | CHMP3 |
| chr18 | 47,007,548 | 47,018,935 | RPL17 | C18orf32 |
| chrX | 100,645,878 | 100,669,128 | RPL36A | HNRNPH2 |
| chr6 | 34,254,973 | 34,393,902 | RPS10 | NUDT3 |
| chr11 | 18,252,902 | 18,270,221 | SAA2 | SAA4 |
| chr15 | 80,135,889 | 80,215,448 | ST20 | MTHFS |
| chr2 | 48,757,064 | 49,003,656 | STON1 | GTF2A1L |
| chr14 | 70,791,798 | 70,883,807 | SYNJ2BP | COX16 |
| chr20 | 35,202,957 | 35,240,960 | TGIF2 | C20orf24 |
| chr5 | 114,914,339 | 114,961,876 | TMED7 | TICAM2 |
| chr3 | 52,867,131 | 52,876,976 | TMEM110 | MUSTN1 |
| chr20 | 48,697,661 | 48,770,335 | TMEM189 | UBE2V1 |
| chr1 | 95,699,711 | 95,710,509 | TMEM56 | RWDD3 |
| chr1 | 151,129,105 | 151,142,773 | TNFAIP8L2 | SCNM1 |
| chr17 | 7,452,375 | 7,464,925 | TNFSF12 | TNFSF13 |
| chr6 | 1,627,064 | 1,644,613 | TRIM39 | RPP21 |
| chr11 | 5,617,865 | 5,665,625 | TRIM6 | TRIM34 |
| chr17 | 15,339,332 | 15,466,945 | TVP23C | CDRT4 |
| chr7 | 43,906,157 | 43,946,231 | URGCP | MRPS24 |
| chr8 | 124,238,429 | 124,286,727 | ZHX1 | C8ORF76 |
| chr19 | 9,435,371 | 9,493,293 | ZNF559 | ZNF177 |
| chr19 | 53,430,388 | 53,466,164 | ZNF816 | ZNF321P |
